# From the ground up: understanding the developing infrastructure and resources of 3D printing facilities in hospital-based settings

**DOI:** 10.1186/s41205-022-00147-7

**Published:** 2022-07-11

**Authors:** Kristy M. Shine, Lauren Schlegel, Michelle Ho, Kaitlyn Boyd, Robert Pugliese

**Affiliations:** 1 Health Design Lab, Thomas Jefferson University, Philadelphia, USA; 2 Sidney Kimmel Medical College, Thomas Jefferson University, Philadelphia, USA; 3 Department of Emergency Medicine, Thomas Jefferson University, Philadelphia, USA; 4 Department of Medicine, Pennsylvania Hospital, University of Pennsylvania, Philadelphia, USA; 5 College of Engineering, Drexel University, Philadelphia, USA; 6 Innovation Pillar, Thomas Jefferson University, Philadelphia, USA

**Keywords:** 3D printing, Additive manufacturing, Hospital, Healthcare, Infrastructure, Survey

## Abstract

**Background:**

3D printing is a popular technology in many industries secondary to its ability to rapidly produce inexpensive, high fidelity models/products, mainly through layer-by-layer fusion of various substrate materials. In healthcare, 3D printing has garnered interest for its applications in surgery, simulation, education, and medical device development, and 3D printing facilities are now being integrated into hospital-based settings. Yet, little is known regarding the leadership, resources, outputs, and role of these new onsite entities.

**Methods:**

The purpose of this research was to survey features of North American hospital-based 3D printing facilities to understand their design and utility in anticipation of future expansion. Hospital-based 3D printing labs were recruited through online special interest groups to participate via survey response. Anonymous, voluntary data were collected from 21 facilities over 9 weeks and reported/analyzed in aggregate.

**Results:**

Of the respondents, > 50% were founded in the past 5 years and 80% in the past decade, indicating recent and rapid growth of such facilities. Labs were most commonly found within large, university-affiliated hospitals/health systems with administration frequently, but not exclusively, through radiology departments, which was shown to enhance collaboration. All groups reported collaborating with other medical specialties/departments and image segmentation as part of the workflow, showing widespread interest in high fidelity, personalized medicine applications. Lab leadership was most often multidisciplinary, with physicians present on nearly all leadership teams. Budgets, personnel, and outputs varied among groups, however, all groups reported engagement in multiple 3D printing applications.

**Conclusion:**

This preliminary study provides a foundation for understanding the unique nature of hospital-based 3D printing labs. While there is much to learn about such in-house facilities, the data obtained reveal important baseline characteristics. Further research is indicated to validate these early findings and create a detailed picture of the developing infrastructure of 3D printing in healthcare settings.

**Supplementary Information:**

The online version contains supplementary material available at 10.1186/s41205-022-00147-7.

## Background

3D printing, also known as additive manufacturing, is the process by which modeling data can be rendered into physical structures through the layer-by-layer fusion of a variety of materials, allowing for the production of 3D forms with complex geometries. A broad range of printing technologies exist, including powder bed fusion, vat photopolymerization, material extrusion, and material jetting, while materials span from metals and concrete to plastics and biomolecules [1–3]. Substrates and printing techniques continue to be developed and enhanced, improving the fidelity of 3D-printed models and fine-tuning their mechanical properties.

The ability to produce highly customizable products makes 3D printing particularly well suited to the development of personalized medicine across specialties. 3D printing has been used for education, clinical decision making, procedural augmentation, and adaptive devices in addition to surgical contouring, surgical guides, splints, and implants [4, 5]. Each specialty has developed unique ways of utilizing 3D printing to meet the demands of that particular field [6, 7]. Surgeons who previously relied on limited and often challenging interpretation of 2D images and 3D reconstructions for surgical planning can now utilize personalized 3D printed models derived from patient-specific imaging leading to increased confidence in the OR [2, 3, 8, 9]. Numerous other studies have been conducted with a focus on education, helping patients to understand complex anatomy and providing medical students/residents opportunities to practice difficult procedures [10–13]. Thus, 3D printing applications in healthcare are vast, ranging from surgical and clinical decision making to trainee and patient education to medical devices, to guides and adaptive technologies. Of note, the Food and Drug Administration is still developing regulatory policy for many applications of 3D printing, but certain applications such as medical or surgical device production, fall under other regulatory policies.

As clinicians and surgeons continue to incorporate 3D printed technologies in their clinical research and practice workflows, 3D printing laboratories are now appearing beyond the engineering and manufacturing sectors in hospital-based settings as well. However, there is currently no central oversight or organization of these hospital-based 3D printing facilities. While substantial knowledge exists on applications of 3D printing in healthcare, there is limited data on the infrastructure of the facilities themselves and their resources (e.g. leadership structure/personnel, budgetary and equipment resources, and outputs). The purpose of this survey research was to identify the practice settings, locations, personnel, and resources of such facilities to gain an understanding of the characteristics that define onsite healthcare-associated 3D printing labs.

## Methods

### 3D printing infrastructure/resource survey generation

A study team was formed to generate a survey characterizing 3D printing laboratories/facilities in healthcare systems. The study team consisted of individuals familiar with conducting research/clinical projects using 3D printing models in the healthcare setting and included a 3D printing technologist, engineer/medical student, 3D printing laboratory manager/innovation specialist, and physician scientist/engineer. Through consensus discussions, the team identified core areas of interest for the survey, including: hospital system/setting characteristics, healthcare-associated 3D printing personnel demographics and training methodologies, laboratory/facility characteristics including equipment and resources, 3D printing applications, printing outputs, and dissemination of 3D printing knowledge. Based on these topics, team members crafted individual survey questions that were edited by consensus to achieve a final survey of 25 questions (Additional file 1). Survey response format was varied and included free text, multiple choice, and multiple selection questions. Participants were allowed to skip questions which they did not feel comfortable answering or for which they did not have the information available.

### Survey distribution and data collection

The study cohort was identified as 3D printing facilities in hospitals/healthcare systems primarily in the United States for the purposes of distribution of the survey in the English language. Because no registry of such facilities exist, the Radiological Society of North America’s 3D printing Special Interest Group message board and Linkedin 3D Printing in Hospitals and Medical Additive Manufacturing & 3D Printing message boards were used to contact potential survey participants. The survey was distributed electronically accompanied by an introductory message and subject recruitment letter detailing the purpose of the study (Additional file 2). Participation was voluntary and anonymous and consent was implied through participation. Survey responses were limited to one survey per 3D printing facility and data was collected over a 9 week period in the fall of 2020.

### Data storage and analysis

Survey response data was stored anonymously and securely using a REDCap database [14, 15]. For free text answers that were returned as non-discrete or a range (e.g. 25+, 10–15), the numeric value stated and range maximum were used for reporting purposes, respectively. Statistical analysis was performed using RStudio software [16]. Given nonparametric data, Wilcoxon rank sum test and Spearman’s rank correlation coefficient tests were used in data analysis. Data analysis was conducted in conjunction with a statistician. All portions of the project were approved by the Institutional Review Board.

## Results

### General survey response

Twenty-five survey responses were received over the data collection period. Of the responses, 4 were received from international facilities outside of North America and were excluded, leaving 21 survey respondents in the data set. Although respondents were not required to complete all questions in the survey, all but one respondent completed greater than 95% of the questions posed. For most survey questions, 18–21 responses were received. Annual 3D printing budget was the exception, with only 9 responses.

### Geographic distribution of 3D printing facilities

Of the 21 North American respondents in the data set, 19 reported their 3D printing facility’s location as in the United States, 1 in Canada and 1 in Mexico. Of US respondents, 8 were located in the Northeast, 7 in the Midwest, 4 in the South, and 0 in the West. Pennsylvania was the most frequent state identified with 5 3D printing facilities.

### Hospital-based environments

**Fig. 1:**
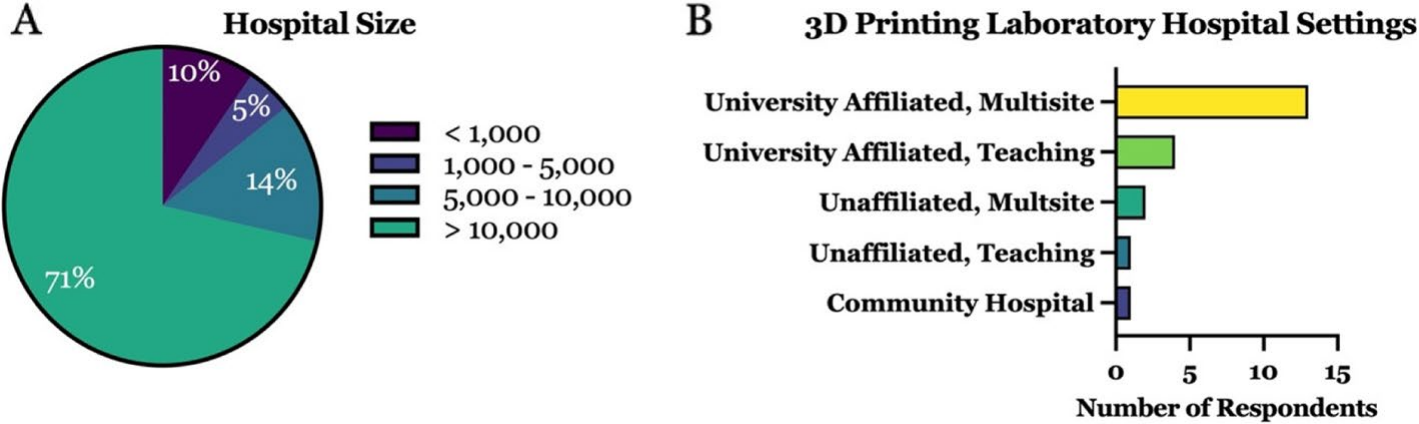
Characterization of hospital-based 3D printing laboratory environments by **A** size of healthcare institution (i.e. total number of employees), and **B** type of institution

The majority of respondents (71%) reported their 3D printing facility was housed within a large healthcare system, notably at institutions with greater than 10,000 employees (Fig. 1A). 81% (17/21) reported being located in a university-affiliated teaching hospital or multisite hospital system, only 4 labs reported no university affiliation, and only one of the groups responding self-identified as community-based (Fig. 1B). No respondents selected their practice setting as a specialty care tertiary, urban acute care, rural acute care, government/military, or rehabilitation hospital, nor selected ‘other’. In terms of distribution within a hospital or healthcare system, 9/21 vs. 12/21 of the survey respondents reported having a multiple site versus single laboratory location, respectively, indicating a split among respondents.

### Establishment and administration

**Fig. 2:**
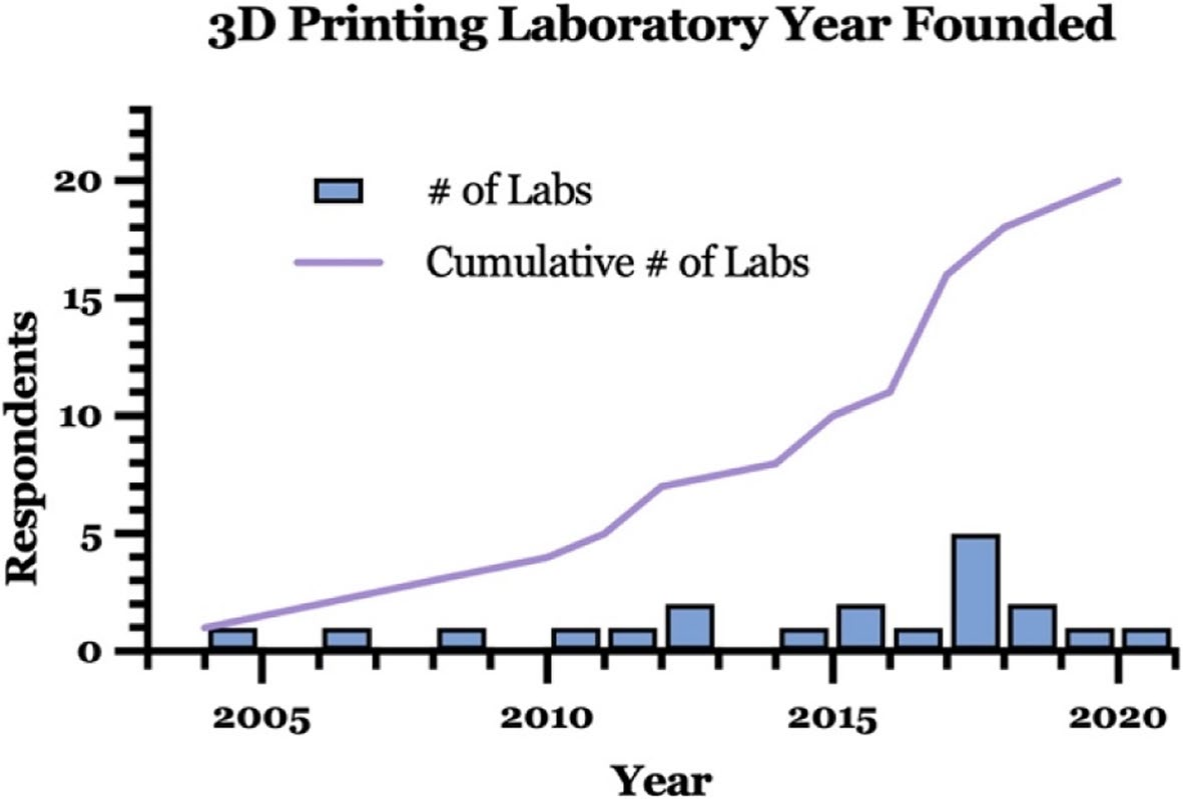
Respondent hospital-based 3D printing laboratory founding years

**Fig. 3:**
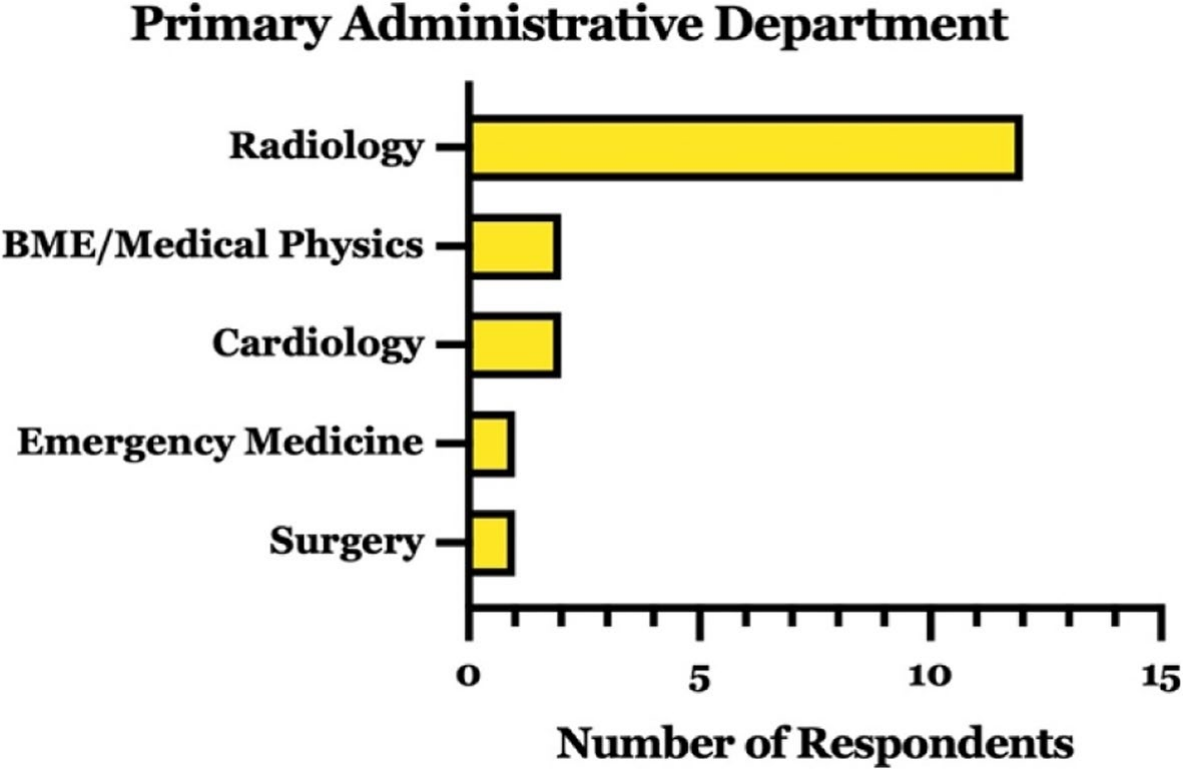
Characterization of 3D printing laboratory department of administration

The earliest reported 3D printing facility in our survey was established in 2004, with 57% of respondents reporting their individual facility opening in the 5 year period prior to the study collection period and 80% in the previous decade (Fig. 2). Eighteen respondents identified their 3D printing facility being administered through a single department, one reported co-departmental administration (without identifying a primary affiliation), and one reported no affiliation (Fig. 3). Among those with an identified primary administrative department, radiology was the most commonly cited (67%). Of those administered outside of radiology, a variety of departments were reported.

### Collaboration and funding

Respondent 3D printing facilities reported collaboration with a large number of other departments, on average collaborating with 10.2 ± 7.5 other departments (mean ± SD) (Table 1). Administration exclusively through radiology versus another department identified had a significant effect on the number of reported collaborators, 13.3 ± 2.3 versus 5.9 ± 1.3 (mean ± SEM) respectively (*p* = 0.017, Wilcoxon rank-sum test). Whereas having multiple 3D printing locations within a hospital or health system did not correlate with increased number of collaborators (*p* = 0.30, Spearman’s rank correlation coefficient test).

**Table 1:** Characterization of 3D printing laboratory collaborating departments. Note: Several respondents listed additional departments in the “other” category, which are included here

Collaborating Departments	Respondents	“Other” Departments	Respondents
Anesthesiology	8	Breast Surgery	1
Cardiology	17	Child Life	1
Dermatology	3	CT/CV/Thoracic Surgery	2
General Surgery	15	Endocrinology/Metabolic	2
Nephrology	6	Fetal Surgery	1
Neurology	9	Gastroenterology	1
Neurosurgery	14	Genitourinary	1
OB/GYN	10	Graduate Med-Ed	1
Ophthalmology	4	Hepatobiliary Surgery	1
Oncology	13	Nuclear Medicine	1
Orthopedics	18	OMFS/Prosthodontics	2
Otolaryngology/ENT	13	Pathology	1
Pediatric Surgery	15	Pediatrics	1
Radiation Oncology	5	Plastic Surgery	4
Rheumatology	1	Podiatry	1
Urology	14	Pulm/Critical Care	2
Other	11	Radiology/IR/Imaging	4
		Simulation/Safety	1
		Transplant Surgery	1
		Vascular Surgery	2

Of the total respondents, only 9/21 facilities chose to disclose their annual operating budget. Two facilities reported no specific budget, while the remaining 7 3D printing groups reported budgets ranging from $5000 to $3,000,000. Of those with a nonzero budget, the average was $545,000 ± 1,089,797 (mean ± standard deviation) and the median budget was $100,000, reflecting high variation in the responses.

**Fig. 4:**
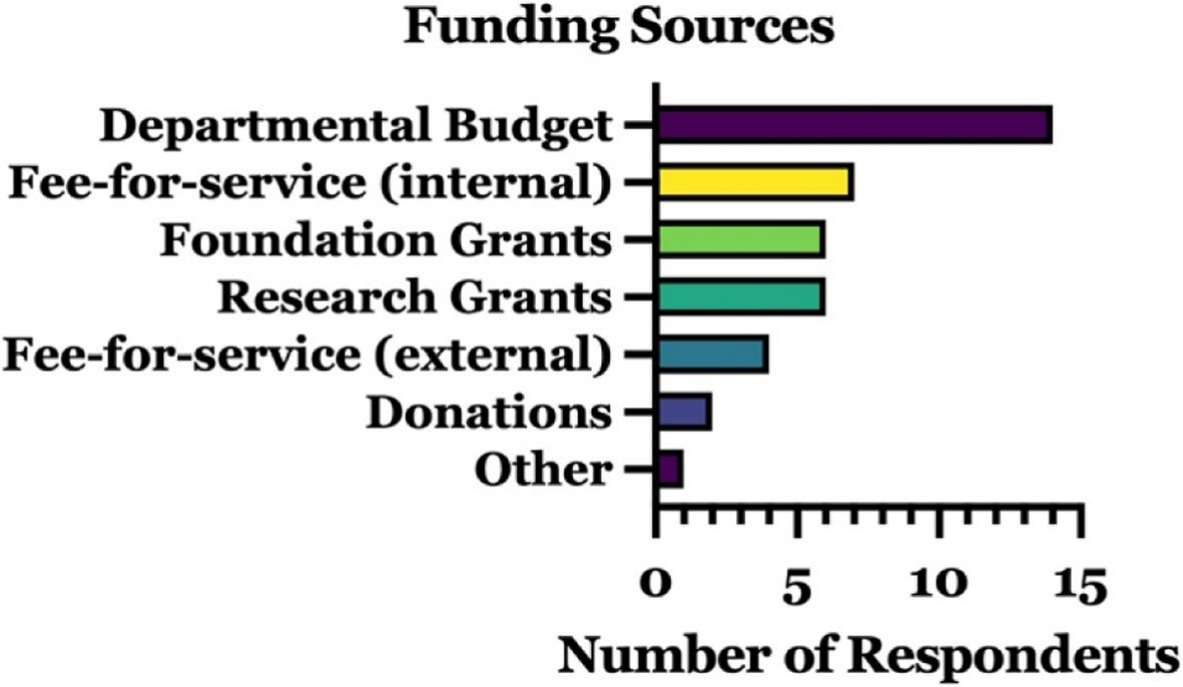
Characterization of 3D printing laboratory funding by source

Conversely, all 21 respondents reported on types of funding, including those who reported no specific budget. Overall, 12/21 facilities reported utilizing more than one type of funding annually, with an average of 1.9 ± 1.1 (mean ± SD) and median of 2 funding sources (Fig. 4). Departmental funds, grants (research or foundation), and fee-for-service models (internal or external) were used by 14, 12, and 11 of the reporting facilities, respectively, indicating no dominant funding paradigm. Donations were reported by only 2 laboratories and one facility reported ‘other’, citing medical billing as a funding source. Labs reported research versus foundation grants with equal frequency whereas internal fee-for-service payments were slightly more frequent than external contracts.

### Personnel

**Fig. 5:**
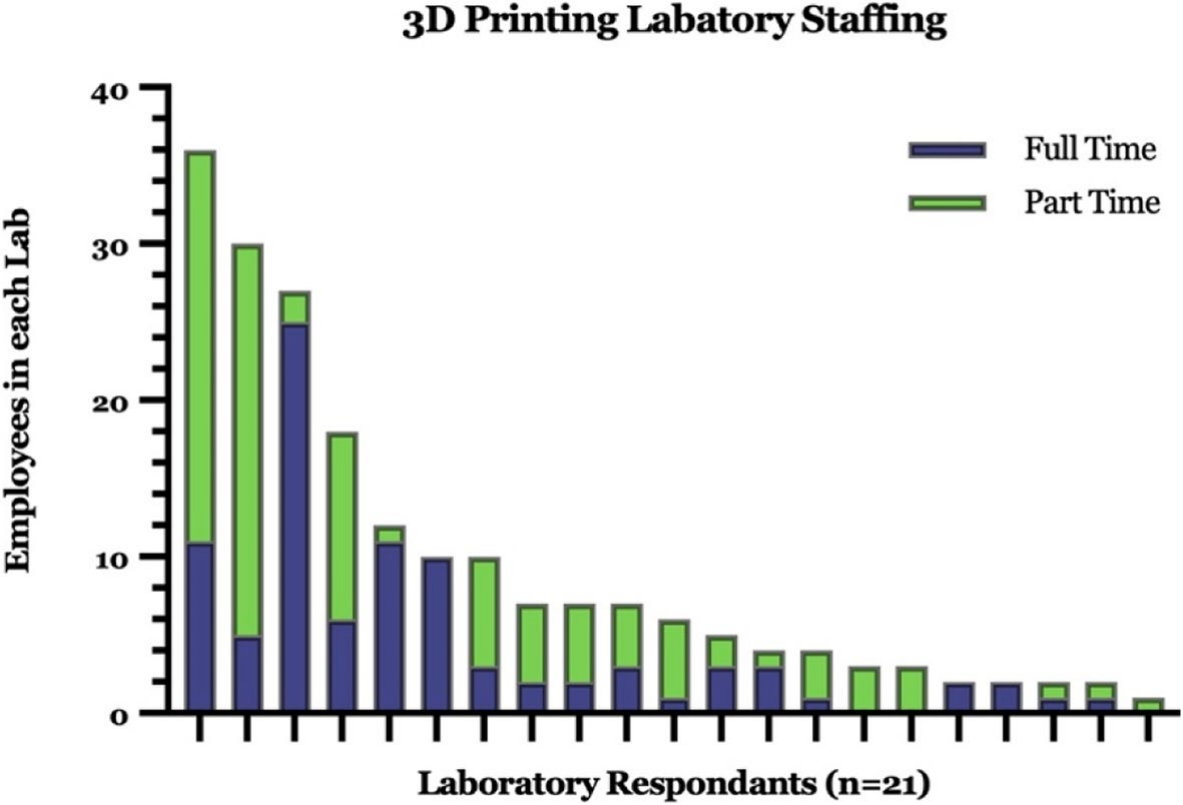
Characterization of 3D printing laboratory staff including full and part-time employees

**Fig. 6:**
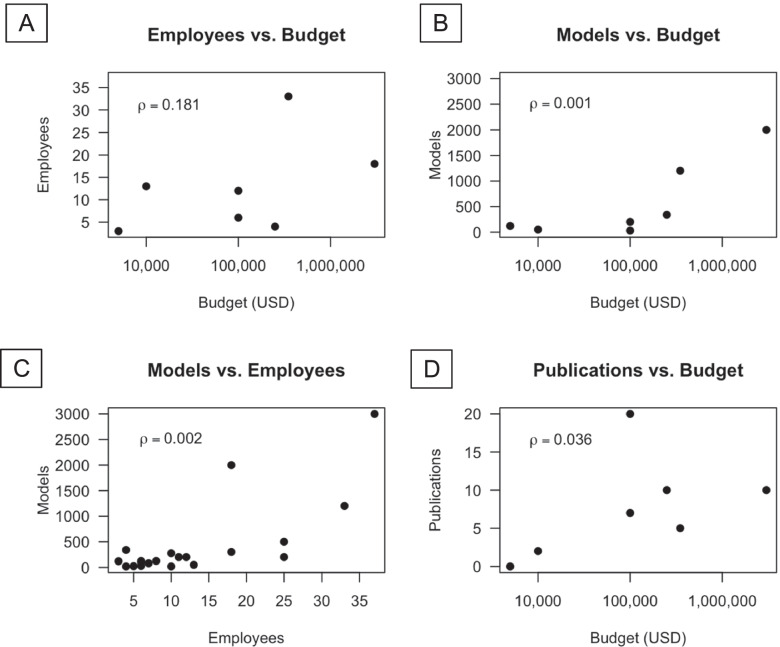
Characterization of 3D printing laboratory **A** employees vs. annual budget, **B** annual models vs. budget, **C** annual models vs. employees, and **D** publications vs. annual budget

3D printing facilities reported a variable number of employees. Approximately 198 individuals were identified as working in 3D printing facilities across the 21 North American respondents. The distribution of full vs. part-time employees is shown in Fig. 5. Notably, there were 4 labs that employed more than 50% of the total reported employees. There was no correlation between annual operating budget and its number of employees (Fig. 6A).

Training of employees was most commonly performed through in-house programs (*n* = 19) rather than outsourced virtual or in-person programs. For in-house training, only 5 facilities reported using a formal training program, whereas 3 facilities reported a mix of formal/informal and 11 reported informal/observational training only.

Of the respondents, 15/20 (75%) reported multidisciplinary leadership teams with 13 of those 15 (87%) having physicians as part of the leadership group. Engineers were the second most frequent profession on the multidisciplinary leadership teams (10 respondents) and students were involved in leadership teams for a limited number of groups reporting (6 respondents). For respondents reporting a single profession compromising the leadership team, there were a variety of different disciplines (physician(s), engineer(s), scientist(s), or lab technician(s)). A total of 15/20 (75%) respondents reported physicians as part of the leadership team.

### Workflow

All 21 groups reported imaging data segmentation as part of their workflow. Two-thirds of the respondents (67%) reported having multiple types of employees performing segmentation. In team-based settings (*n* = 14 groups), physicians were present in 9, engineers in 10, lab technicians/technologists in 7, and students in 5 groups. For groups with only one personnel type performing segmentation (*n* = 7), physicians were the sole individual(s) performing segmentation in 2, engineers in 0, lab technicians/technologists in 4, and students in 1 group(s). Overall, regardless of multidisciplinary vs uniform teams, image segmentation was well distributed among physicians (11/21), engineers (10/21), and lab technicians/technologists (11/21), whereas fewer groups reported student participation (6/21).

Nineteen groups reported 3D printing as an included element of their workflow. One group reported outsourcing 3D printing post segmentation, and one group did not respond. Within laboratories conducting in-house 3D printing, 15/19 respondents (79%) reported having multiple types of employees operating the 3D printers. In team-based 3D printing settings (*n* = 15), physicians were present in 7, engineers in 10, lab technicians/technologists in 8, and students in 7 groups. For groups with only one personnel type operating the 3D printers (*n* = 4), physicians were the sole individual(s) performing printing operation in 1, engineers in 1, lab technicians/technologists in 2, and students in 0 group(s).

### Equipment and resources

**Fig. 7:**
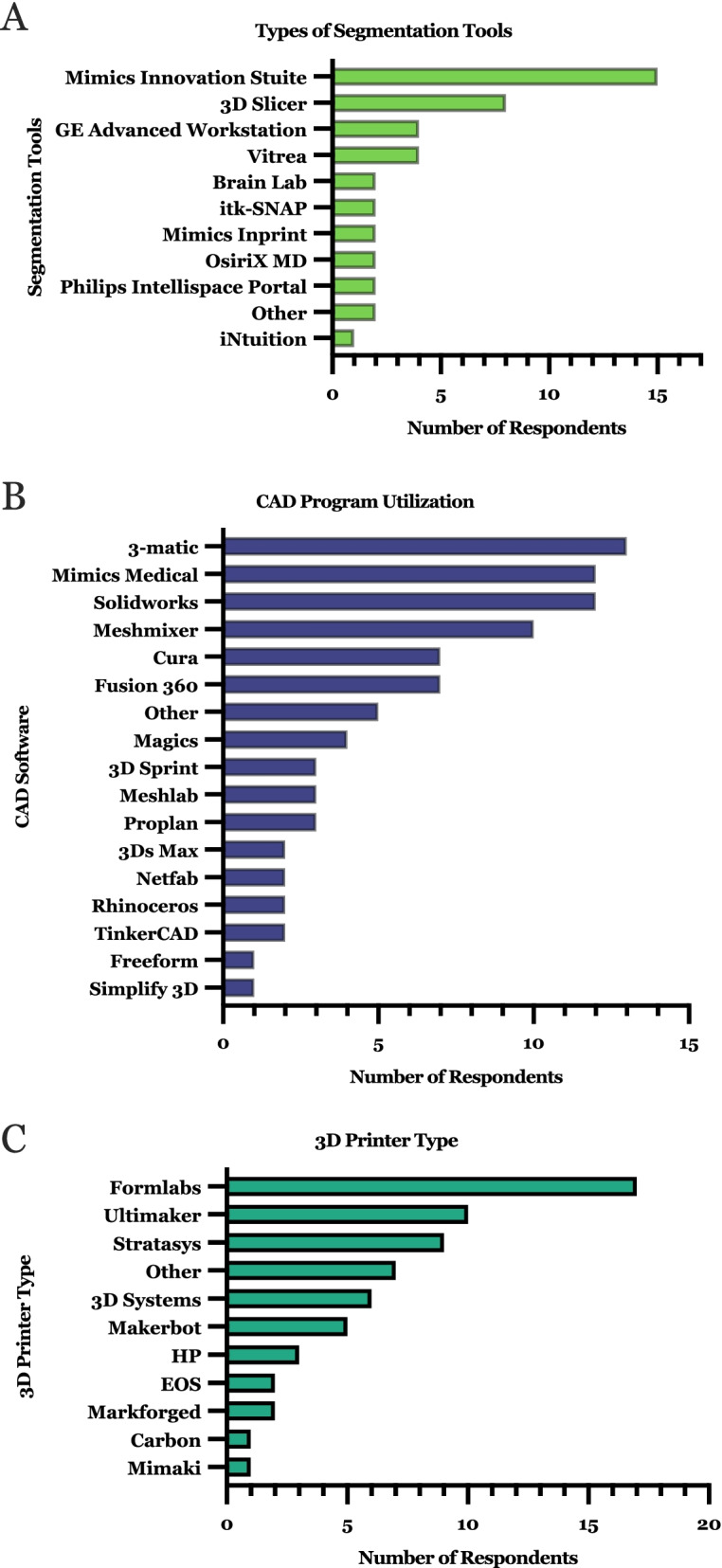
Characterization of 3D printing laboratory tools including **A** segmentation software, **B** CAD software, and **C** printer hardware. Note: for segmentation and CAD software, no respondents selected Seg3D, D2P, Amira, or 4 DICOM, and Biomesh3D, Dolphin, or Onshape, respectively

Most respondent facilities used more than one segmentation software (13/20), whereas only 7/20 used more than one CAD program in their workflows. For imaging segmentation, Mimics Innovation Suite and 3D Slicer were the most commonly used software programs among the respondents (Fig. 7A). For CAD/processing, the most commonly used programs included 3-matic, Mimics Medical, and Solidworks (Fig. 7B). Similarly, respondent facilities reported using a variety of 3D printers in their individual facilities, with an average of 3.3 ± 2.1 (mean ± SD) printer types (Fig. 7C). Formlabs were the most commonly used printers followed by Ultimaker models. There was no significant association between the budget and number of printers reported by each lab (*p* = 0.159, Spearman’s rank correlation coefficient test).

### Applications of 3D prints

**Fig. 8:**
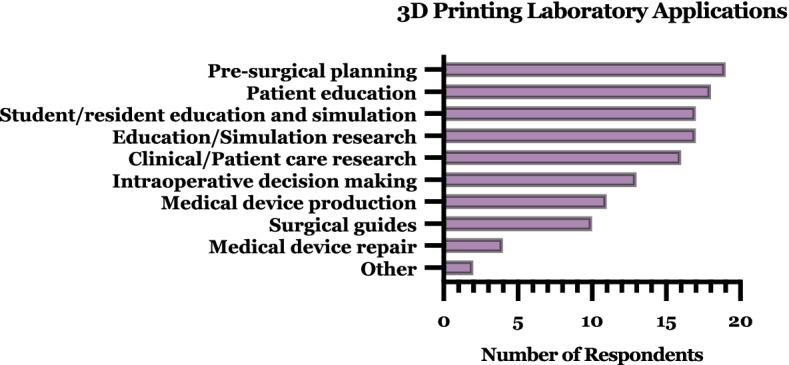
Characterization of 3D printing laboratory output applications. Note: “other” uses specified in free text included promotion and dental models

There was no dominant use for 3D printing among the survey respondents (Fig. 8). All respondent laboratories reported more than one application for their 3D printed projects, with an average of 6.4 ± 1.7 (mean ± SD) applications. At least one or more surgical application (i.e. presurgical planning, intraoperative decision making, or surgical guide production) and one or more educational applications (i.e. patient education, student/resident education and simulation, or education/simulation research) were reported by every survey respondent (*n* = 20). All but one of the respondents also reported at least one research application (i.e. education/simulation research or clinical/patient care research). Approximately half of the groups (11/20) reported also using 3D printing for a medical device application (i.e. production or repair). Only 2 groups reported an ‘other’ application not captured in the survey response selection options - promotion and dental models. Across facilities, approximately half (9 of 20) also had virtual reality capabilities, showing interest in 3D environments outside of printed work.

The majority of 3D printing facilities (80%) reported making 350 or fewer 3D printed constructs annually. Only 4 facilities reported making more, ranging from 500 to 3000 pieces per year. There was a strong correlation between annual budget and number of models produced (*p* = 0.001, Spearman’s rank correlation coefficient test, Fig. 6B) and number of employees and number of models produced (*p* = 0.002, Spearman’s rank correlation coefficient test, Fig. 6C); However, there was no significant correlation between budget and number of applications.

### Scholarly outputs

17 of 19 responding 3D printing groups reported publishing their work with a total of 112 papers published, with an average of 6.6 ± 5.8 (mean ± SD), median of 5, and range of 1–20 publications annually among the survey respondents. In total, 112 publications were reported. Notably, 36% of publications were reported by just 2 of the responding facilities and 63% from the top 5 most prolific groups. Publication impact factors or other quality rankings were not assessed in this study. There was a significant correlation between annual budget and number of publications (*p* = 0.036, Spearman’s rank correlation coefficient test, Fig. 6D).

Nearly all of the respondent groups (19 of 20) reported their staff attending and/or presenting at medical conferences annually, 12 of 20 reported attending and/or presenting at additive manufacturing conferences, and 9 of 20 reported attending innovation conferences. ‘Other’ types of conferences attended included dental, engineering, and medical illustration.

## Discussion

3D printing is increasingly being utilized in healthcare applications, prompting hospital systems to bring this technology and its relevant workflows in-house. Yet, little is known regarding the network of such hospital-based facilities nor their operating characteristics, personnel, resources, or impact, highlighting the gap our preliminary research sought to address. Obtaining such information may allow for an understanding of the conditions that favor the integration and use of 3D printing in medical workflows, thus opening opportunities for research expansion, new medical education tools, and enhanced patient care. To our knowledge, this is the first survey to assess the infrastructure and resources of 3D printing facilities in North American hospital-based settings.

In consideration of survey participation and potentially sensitive information disclosures (e.g. budgets, productivity, etc.), we felt it important to make this introductory survey voluntary and anonymous Moreover, respondents were allowed to skip questions for which they did have data or feel comfortable answering. As a result of our current approach, the strength of our survey rests in the scope and depth of detail it elucidated in its anonymous form. Indeed, all but one respondent completed greater than 95% of the survey questions posed.

Among our survey respondents, our results show that hospital-based 3D printing laboratories are most frequently associated with large university affiliated and teaching hospitals/healthcare systems rather than non-university or community facilities. Such laboratories also nearly exclusively favored the use of in-house staff training versus external options suggesting such facilities develop onsite expertise. We hypothesize that these observations are a result of current medical 3D printing applications existing largely in the research and development phase, requiring more resource rich institutions, rather than in the clinical application phase secondary to the infancy of the field at large. Indeed, our results showed most 3D printing labs surveyed were established only in the last decade and none greater than 17 years prior to the data collection period.

Radiology was the department most frequently cited as the home department for hospital-based 3D printing labs in the dataset, likely reflecting the recruitment process. Reported interdepartmental collaboration was also high among survey respondents with an average of 10–11 departmental partners per group in keeping with the majority of respondent facilities operating within a specialty noted to interact with many other specialties. A third of respondents reported another primary department suggesting at least some diversity in responses captured by the dataset despite inherent bias in the recruitment process. Moreover, individuals comprising lab personnel were similarly diverse including physicians, engineers, technicians, and students. Physicians and engineers were the group most actively involved in lab leadership, whereas specific workflow activities were more evenly split among these groups and laboratory technicians.

Despite overwhelmingly similar institutional settings and propensity to collaborate, other baseline characteristics among facilities varied greatly, including employee totals and budgets. Total lab personnel varied 36-fold between the lowest and highest values reported and just 4 facilities employed nearly 60% of all reported hospital-based 3D printing lab staff. Budgets varied even more, ranging from none to millions of dollars. Hardware and software used by facilities also varied to a large extent as did printer models. Finally, outputs in terms of numbers of prints and publications and conference involvement were variable among the groups. There was a correlation between annual budget and number of constructs, as well as budget and publications produced. However, the number of employees did not appear to significantly alter publications.While providing initial insights into the developing infrastructure and resources of hospital-based 3D printing facilities, our work is based on a small dataset from largely US participants based on recruitment strategy. In this study, we attempted to identify and contact 3D printing hospital-based facilities through widely subscribed 3D printing special interest group message boards, given no pre-existing central databases for such facilities. However, such message boards target largely a North American, English-speaking audience precluding participation by much of the international community and facilities unaffiliated with the message boards. Message board recruitment also prevented survey response rate determination, so it is unclear how many facilities exposed to our recruitment opportunity opted not to participate. Therefore, we suspect, and consider it highly likely, that there are other hospital-based 3D printing facilities not captured in our dataset. Moreover, there are likely smaller 3D printing groups in non-hospital, but medically-based settings (e.g. rehabilitation facilities, outpatient offices, surgi-centers) that were not captured.

Using a specialty-specific (i.e. radiology) message board, as expected, our data showed the majority of facilities responding were administered through radiology. However, given approximately one third of facilities responding were not administered through radiology, there was noted be be at least some diversity of affiliated specialties among respondent message board users. While the recruitment strategy is an identified limitation, it highlights the critical gap in organization for hospital-based and healthcare-associated 3D printing practices. Despite foundational work being done by RSNA, technology/engineering associations, and informal user-led groups, we were not able to identify an organization through which to recruit participants that represents the diversity of focus, personnel, and practice of hospital-based and healthcare-associated 3D printing practices limiting our scope.

The dataset was collected over a short period, which may have excluded infrequent message board viewers and contributed to overall reduced participation. It is also important to acknowledge that the study data collection period occurred during the covid-19 pandemic, during which many hospital systems were temporarily suspending operations of non-essential facilities and redirecting resources to support increased clinical demands. Given the variation in international, state, and local guidelines, it is unclear to what extent this prevented individual or regional hospital-based 3D printing facilities from viewing and responding to the recruitment letter given the short data collection period.

## Conclusion

This preliminary study provides a foundation for understanding the unique nature of hospital-based 3D printing labs. While there is still much to learn about such facilities, and those in other smaller healthcare-associated settings, the survey data presented reveals important baseline characteristics. Further research is indicated to continue to validate these findings, expand discovery of other existing facilities, and create a more detailed picture of the developing infrastructure of 3D printing in the healthcare setting.

## Supplementary Information

**Additional file 1.**

**Additional file 2.**

## Data Availability

Survey questions (“Additional file 1.pdf” – Understanding 3D Printing Infrastructure and Resources Survey) and study recruitment letter (“Additional file 2.docx” – Special Interest Group Posting/Subject Recruitment Letter) are included. The dataset supporting the conclusions of this article is available to reviewers upon request.
