# Supplementary material for: From the ground up: understanding the developing infrastructure and resources of 3D printing facilities in hospital-based settings
Source: 3D Print Med. 2022 Jul 11;8:21. doi: 10.1186/s41205-022-00147-7 (PMC9275538; doi:10.1186/s41205-022-00147-7)
Supplement: Supplementary file 1 — Additional file 1. [file 41205_2022_147_MOESM1_ESM.pdf]

Understanding 3D Printing Infrastructure and Resources

In what country is your institution? __________________________________

In what province/state/district is your institution?

- ◯ Alabama
- ◯ Alaska
- ◯ Arizona
- ◯ Arkansas
- ◯ California
- ◯ Colorado
- ◯ Connecticut
- ◯ Delaware
- ◯ Florida
- ◯ Georgia
- ◯ Hawaii
- ◯ Idaho
- ◯ Illinois
- ◯ Indiana
- ◯ Iowa
- ◯ Kansas
- ◯ Kentucky
- ◯ Louisiana
- ◯ Maine
- ◯ Maryland
- ◯ Massachusetts
- ◯ Michigan
- ◯ Minnesota
- ◯ Mississippi
- ◯ Missouri
- ◯ Montana
- ◯ Nebraska
- ◯ Nevada
- ◯ New Hampshire
- ◯ New Jersey
- ◯ New Mexico
- ◯ New York
- ◯ North Carolina
- ◯ North Dakota
- ◯ Ohio
- ◯ Oklahoma
- ◯ Oregon
- ◯ Pennsylvania
- ◯ Rhode Island
- ◯ South Carolina
- ◯ South Dakota
- ◯ Tennessee
- ◯ Texas
- ◯ Utah
- ◯ Vermont
- ◯ Virginia
- ◯ Washington
- ◯ West Virginia
- ◯ Wisconsin
- ◯ Wyoming
- ◯ District of Columbia
- ◯ Puerto Rico
- ◯ US Virgin Islands
- ◯ Alberta
- ◯ British Columbia
- ◯ Manitoba
- ◯ New Brunswick
- ◯ Newfoundland and Labrador
- ◯ Northwest Territories
- ◯ Nova Scotia
- ◯ Nunavut
- ◯ Other

Please specify.	__________________________
How large is your institution?	◯ less than 1,000 employees<br>◯ 1,000 - 5,000 employees<br>◯ 5,000 - 10,000 employees<br>◯ more than 10, 000 employees
What type of institution does your 3D printing facility(s) serve?	◯ Large University Affiliated Health System with Multiple Hospitals<br>◯ Large Unaffiliated Health System with Multiple Hospitals<br>◯ University Affiliated Teaching Hospital<br>◯ Unaffiliated Teaching Hospital<br>◯ Specialty Care Tertiary Hospital<br>◯ Urban Acute Care Hospital<br>◯ Rural Acute Care Hospital<br>◯ Community Hospital<br>◯ Government/Military Hospital<br>◯ Rehabilitation Hospital<br>◯ Other
Please specify.	__________________________
Does your institution have a centralized, medical 3D printing lab/facility(s)?	◯ Yes<br>◯ No
Does your organization have distributed 3D printing locations across multiple areas?	◯ Yes<br>◯ No
How many sites?	__________________________
In what year was your 3D printing facility(s)/service founded?	__________________________
By what department is your 3D printing facility(s) administered?	__________________________
With what department(s) does your 3D printing facility(s) collaborate?	☐ Anesthesiology<br>☐ Cardiology<br>☐ Dermatology<br>☐ General Surgery<br>☐ Nephrology<br>☐ Neurology<br>☐ Neurosurgery<br>☐ OB/GYN<br>☐ Opthomology<br>☐ Oncology<br>☐ Orthopedics<br>☐ Otolaryngology/ENT<br>☐ Pediatric Surgery<br>☐ Radiation Oncology<br>☐ Rheumatology<br>☐ Urology<br>☐ Other<br>(Check all that apply)

Please specify.	______________________
Which areas does your 3D printing facility(s) support?	☐ Pre-surgical planning<br>☐ Intraoperative decision making<br>☐ Surgical guides<br>☐ Patient education<br>☐ Student/resident education and simulation<br>☐ Medical device production<br>☐ Medical device repair<br>☐ Clinical/Patient care research<br>☐ Education/Simulation research<br>☐ Other<br>(Check all that apply)
Please specify.	______________________
Who comprises your leadership team at your 3D printing facility(s)?	☐ Lab technician(s)<br>☐ Student(s)<br>☐ Physician(s)<br>☐ Engineer(s)<br>☐ Scientist(s)<br>☐ Other<br>(Check all that apply)
Please specify.	______________________
Who operates the equipment in your 3D printing facility(s)?	☐ Lab technician(s)<br>☐ Student(s)<br>☐ Physician(s)<br>☐ Engineer(s)<br>☐ Scientist(s)<br>☐ Other<br>(Check all that apply)
Please specify.	______________________
Who does segmentation (pre-validation) in your 3D printing facility(s)?	☐ Lab technician(s)<br>☐ Student(s)<br>☐ Physician(s)<br>☐ Engineer(s)<br>☐ Scientist(s)<br>☐ Other<br>(Check all that apply)
Please specify.	______________________
How are new users trained in your 3D printing facility(s)?	☐ Formalized In-house Training Curriculum<br>☐ Informal/observational In-house Training<br>☐ Out-sourced Live Training<br>☐ Out-sourced Online Training<br>☐ Other
Please specify.	______________________

How many people work in your 3D printing facility(s) full-time?

- ◯ 1
- ◯ 2
- ◯ 3
- ◯ 4
- ◯ 5
- ◯ 6
- ◯ 7
- ◯ 8
- ◯ 9
- ◯ 10
- ◯ 11
- ◯ 12
- ◯ 13
- ◯ 14
- ◯ 15
- ◯ 16
- ◯ 17
- ◯ 18
- ◯ 19
- ◯ 20
- ◯ 21
- ◯ 22
- ◯ 23
- ◯ 24
- ◯ 25
- ◯ 25+

How many people work in your 3D printing facility(s) part-time?

- ◯ 1
- ◯ 2
- ◯ 3
- ◯ 4
- ◯ 5
- ◯ 6
- ◯ 7
- ◯ 8
- ◯ 9
- ◯ 10
- ◯ 11
- ◯ 12
- ◯ 13
- ◯ 14
- ◯ 15
- ◯ 16
- ◯ 17
- ◯ 18
- ◯ 19
- ◯ 20
- ◯ 21
- ◯ 22
- ◯ 23
- ◯ 24
- ◯ 25
- ◯ 25+

What printers does your facility(s) have?	☐ 3D Systems<br>☐ Carbon<br>☐ Envision TEC<br>☐ EOS<br>☐ ExOne<br>☐ Formlabs<br>☐ HP<br>☐ Makerbot<br>☐ Markforged<br>☐ Mimaki<br>☐ Raise 3D<br>☐ Stratasys<br>☐ Ultimaker<br>☐ Other<br>(Check all that apply)
How many 3D Systems printers does your facility(s) have?	______________________
How many Carbon printers does your facility(s) have?	______________________
How many Envision TEC printers does your facility(s) have?	______________________
How many EOS printers does your facility(s) have?	______________________
How many ExOne printers does your facility(s) have?	______________________
How many Formlabs printers does your facility(s) have?	______________________
how many HP printers does your facility(s) have?	______________________
How many Makerbot printers does your facility(s) have?	______________________
How many Markforged printers does your facility(s) have?	______________________
How many Mimaki printers does your facility(s) have?	______________________
How many Raise 3D printers does your facility(s) have?	______________________
How many Stratasys printers does your facility(s) have?	______________________
How many Ultimaker printers does your facility(s) have?	______________________
What other 3D printers does your facility(s) have and how many?	______________________

What CAD software does your facility(s) use?	☐ 3D Sprint<br>☐ 3Ds Max<br>☐ 3-matic<br>☐ Biomesh3D<br>☐ Cura<br>☐ Dolphin<br>☐ Freeform<br>☐ Fusion 360<br>☐ Magics<br>☐ Meshlab<br>☐ Meshmixer<br>☐ Mimics Medical<br>☐ Netfab<br>☐ Onshape<br>☐ Proplan<br>☐ Rhinoceros<br>☐ Simplify 3D<br>☐ Solidworks<br>☐ TinkerCAD<br>☐ Other<br>(Check all that apply)
Please specify.	__________________________________
What segmentation software does your facility(s) use?	☐ 3D Slicer<br>☐ 4 DICOM<br>☐ Amira<br>☐ Brain Lab<br>☐ D2P<br>☐ GE Advanced Workstation<br>☐ iNtuition<br>☐ itk-SNAP<br>☐ Mimics Innovation Stuite<br>☐ Mimics Inprint<br>☐ OsiriX MD<br>☐ Philips Intellispace Portal<br>☐ Seg3D<br>☐ Vitrea<br>☐ Other<br>(Check all that apply)
Please specify.	__________________________________
Does your facility(s) support the use of other advanced visualization technologies such as Virtual Reality, Augmented Reality, or Mixed Reality systems	○ Yes<br>○ No
How is your 3D printing facility(s) funded?	☐ Research Grants<br>☐ Foundation Grants<br>☐ Donations<br>☐ Departmental Budget<br>☐ Fee-for-service (internal departments)<br>☐ Fee-for-service (external entities/sales)<br>☐ Other<br>(Check all that apply)
Please specify.	__________________________________

What is the annual budget of your 3D printing facility(s)?	__________________________________
At which conferences do users of your 3D printing facility(s) attend and/or present?	☐ Medical<br>☐ Additive Manufacturing<br>☐ Innovation<br>☐ Other<br>(Check all that apply)
Please specify.	__________________________________
Approximately how many publications are enabled by your 3D printing facility(s) annually?	__________________________________
Approximately how many models does your 3d printing facility(s) make annually?	__________________________________
