# Supplementary material for: From the ground up: understanding the developing infrastructure and resources of 3D printing facilities in hospital-based settings
Source: 3D Print Med. 2022 Jul 11;8:21. doi: 10.1186/s41205-022-00147-7 (PMC9275538; doi:10.1186/s41205-022-00147-7)
Supplement: Supplementary file 2 — Additional file 2. [file 41205_2022_147_MOESM2_ESM.docx]

Special Interest Group Posting

Dear Colleagues,

I hope you will consider participating in a national survey we are conducting on 3D printing infrastructure and resources available to hospital systems (recruitment letter and link below). This IRB approved study will be open until [**October 13th at 8PM EST]*.** If you have any questions at all, please do reach out to me and/or Dr. Shine and we would be happy to address them.

Sincerely,

Rob Pugliese

Note: Study period was extended to November 30th at 8pm EST secondary to pandemic delays.

# Subject Recruitment Letter

Dear Subject:

Hello, my name is Kristy Shine, MD, PhD. I’m from Thomas Jefferson University’s Health Design Lab and Department of Emergency Medicine.

You are invited to participate in a survey on the 3D printing infrastructure and resources available to hospital systems. The study team requests that only one survey is completed per 3D printing facility.

You will receive no direct benefits from participating in this research study. However, your responses may help us learn more about 3D printing. This will help guide future endeavors in 3D printing in healthcare.

Our study consists of completing an online survey*.* We estimate that this will take about 5 minutes of your time to complete. Your participation in this study will contribute to advancing our understanding of 3D printing resources available at different institutions.

Your participation in this study is entirely voluntary, and you can end your participation, if you wish, at any time.

If you decide to participate in this study, the survey can be found here

<https://redcap.jefferson.edu/surveys/?s=HXDMXXFAJT>

If any question makes you feel uncomfortable, you don’t have to answer it. I also want to assure you that any information you provide will remain strictly confidential. Your name will not be identified or associated with any specific responses, and it will not appear in any published materials which result from this research.

Thank you for volunteering to participate in this study.

Sincerely,

Kristy Shine, MD, PhD

Principle Investigator

Health Design Lab

Thomas Jefferson University

kristy.shine@jefferson.edu
